# Hints to Diagnosis

**Published:** 1856-10

**Authors:** Hyde Salter


### *Hints to Diagnosis. A Clinical Lecture Delivered at the Charing Cross Hospital.*

BY HYDE SALTER, M. D.

GENTLEMEN: In casting about for a suitable subject for this my first clinical lecture to you—something that should be at once general and practical—it has occurred to my mind that I could not choose any thing that would better fulfil these conditions, or be likely to be more useful to you, or be capable of more frequent application and illustration in my after instructions, than laying down for you some simple rules for your guidance in the formation of diagnosis. Such rules, if worth any thing, must be valuable to all, but especially to those whose practical investigation of disease is all before them.

Correctness of diagnosis is an all-important thing: of your practical duties in after life it will constitute a fair half, for the recognition and comprehension of their nature will be a necessary condition of the correct treatment of the cases that will come under your care. I think myself that diagnosing disease is a mental process of no mean order, that it calls into exercise some of the highest faculties of the mind, and that to do it well, requires a mind minute and exact, scrutinizing and inquisitive, retentive, and analytical, capable of generalizing, and close in its reasoning. There is nothing in which the members of our profession differ more from one another than in their way of making a diagnosis. Given, two men, equally well informed in their profession, with an equal substratum of knowledge to start from, the one's diagnosis will be made with twice the ease and certainty, and in half the time of the other's. Some blunder and bungle in a blind sort of way; they strike out here and there like a man fighting in the dark, without any aim or method, and their hitting the right thing is little better than a matter of chance; some on the other hand, address themselves to their work in a way entirely systematic, they do not throw away a single question, or put a purposeless one; there is not one too much, or one too few; they drive each well home: they keep their aim in view with an undeviating steadiness, and follow it up with a pertinacity that nothing can divert or abate: to watch a diagnosis made in this way is one of the greatest treats—I know of few greater; but to listen to one clumsily made is an intolerable infliction; but, worse than this, it is probably erroneous, and, therefore, perilous to your patient; for on a false diagnosis you will hardly raise a correct therapeusis.

Let me, therefore, beg your attention to the following short and simple rules, which I am sure you will find of great assistance to you.

Rule I.—Gain information in all possible ways without, and additional to, asking questions; that is, take the testimony of your senses—of sight hearing, touch, taste, smell; examine well your patient's physiognomy, his expression, his complexion, his general aspect, his manner; observe his physical condition, if there are in it any of those pathognomonic signs that certain diseases hold out; mark the tone of his voice, its strength and weakness, whether there is in it any thing emotional, whether it is hurried or deliberate. You will find the habitual practice of this great advantage to you and the evidence thus acquired of great value, for,

1st. It is a kind of evidence that cannot be sophisticated. Your senses cannot deceive you, your patient may, at least, attempt it, and this evidence may either corroborate his oral testimony or refute it. For example, suppose a man, on a club, professes the sight of one of his eyes to have failed, and so to be unable to follow his employment, and to be suspected of *malingering*, and on seeing him you detect an inequality of his pupils, and a slight ptosis of one of his eyelids, which had escaped the uninitiated; you feel at once that the man is probably speaking the truth. Suppose, on the other hand, that a man, whose symptoms make you suspect threatening delirium tremens, denies having taken any spirits for a week, and you smell his breath strongly of gin, you have not only strong presumptive evidence that your opinion is correct, but that the man's statements must be disregarded. This kind of evidence is, therefore, not only the most unimpeachable in itself, but a valuable check on other testimony.

2d. It gives you a kind of evidence that nothing else will. Some things, definite in themselves, and that the senses can recognize, can yet not be described. Those who have once seen them know what they are, but no others. Such things are sometimes very subtle and delicate, but they are often very important and distinctive, even pathognomonic. Take, for instance, certain physiognomies; take the facies hysterica: I never saw a good description of this, I could not give one myself, but when once recognized, it is very unmistakable, and very characteristic, and might make all the difference in your opinion of any given case. In some instances, as in skin disease, this sense-testimony is the only evidence you require.

3d. It is evidence you can obtain without your patient's consciousness; and this under some circumstances is most important, because in some things the consciousness of the patient introduces a disturbing element, and invalidates the conclusions you would draw. For example, in counting the respiration, if your patient knows what you are about he immediately directs his attention to his breathing, he breathes voluntarily, and, inevitably, unnaturally; the moment the will is introduced into the respiratory act, which ought to be unconscious and involuntary, it becomes unnatural. When, therefore, I count the respiration, in order to divert the patient's attention from what I am doing, I feel the pulse, which he imagines I am counting, and so breathes naturally. So, again, with regard to the pulse, in some cases, especially where there is much emotional disturbance, it is very desirable to ascertain its condition without the patient's consciousness, which would at once derange it. Now, taking the wrist would directly inform him of what is going on; I therefore pass my hand over the forehead, and keep it for a few seconds applied to the temples, as if I were feeling the temperature of the head, while in reality I am ascertaining the state of the pulse from the temporal artery.

4th. In some cases it is the only evidence you can get, as in the case of young children, or those who are reduced by their state of disease for the time to the condition of infants, in the etymological sense of the word, that is, cannot express themselves, as, for instance, from mania, coma, or any other condition interfering with intelligent speech.

5th. It will save you an immense deal of time; you may often by a glance obtain an amount of information that it would take a long time to acquire by question and answer. Practice will render this survey of your patient, and your conclusions from it, wonderfully rapid, and moreover, render them more comprehensive and more infallibly correct; you will be astonished by and by at the almost instinctive correctness of the impressions you will thus acquire; from the slenderness of the data, the unconsciousness of mental process, and the rapidity at which the conclusions are arrived at, they will seem to you like inspirations. As an instance of the saving of time, see how one's work is shortened by chlorotic lips or a yellow conjunctiva.

Now, there are two kinds of such evidence as this—sense-evidence: one is that which is gained without any special process, without the use of any instrument or artificial means, which is immediate, impromptu, and of which the patient is unconscious; the other is that acquired by some instrumental means or special process, as by the stethoscope, by physical examination, the microscope, chemical reagents, etc. It is the former to which I particularly refer.

Rule II.—Lay greater stress on some signs than on others; as, for example,

*a.* On anatomical than on functional disturbances. Sometimes symptoms are discrepant—the tide of anatomical evidence will set in one direction and functional in another; in this case, always give the preference to evidence of an anatomical nature, for anatomical signs are always of more certain import. Functional symptoms have often the value of probability only—they imply; anatomical symptoms have generally the value of certainity—they assert. For example: suppose a patient to have been suffering for a week from all the symptoms of acute pleurisy—intense pain an inch or two below the nipple, inability to take a deep breath, or to lie on that side, intolerance of pressure in the corresponding intercostal space or spaces—but suppose that, on listening, you hear pure and natural respiratory murmur, and a clear absence of all friction-sounds, and that percussion and auscultation give you evidence of the thoacic viscera being in every respect anatomically healthy. Here you have a dilemma; anatomy says one thing, function another—which are you to believe? Anatomy, certainly. Why? Because you know that if the cause of pain had been pleuritis, you must by that time have had some anatomical result of it; its absence, therefore, negatives the supposition: and so you infer that it is rheumatism of the intercostals, or other muscles of that part of the partietes; or neuralgic; or, if in the case of a young woman, possibly hysterical.

*b.* Lay greater stress on some functional symptoms than on others; for example, on tenderness than pain. Suppose a patient were to come to you one day, complaining of colicky pain in the epigastrium and about the umbilicus, passing through to the back, and the next day you find he has tenderness in the right iliac fossa, you know that this last is the real seat of the mischief, and the other merely the situation to which the pain was referred; this is a phenomenon of frequent occurrence in abdominal affections. Again, vomiting has a certain indicativeness, but blood-vomiting a much higher and more distinctive one.

*c.* Distinguish between pathognomonic and indifferent symptoms. The value of indifferent symptoms—i. e. those common to many diseases—is general, that of pothognomonic, special; indifferent symptoms may leave you many alternatives, pathognomonic, none. Fever, for example, is an indifferent symptom, but the several stages of the fever-paroxysm occurring at the same time every other day is pathognomonic; it leaves you no alternative; you know at once what you have to deal with.

Rule III.—Pursue a definite order in your catechisings. The principle on which a diagnosis is made is that of elimination, of exclusion, or, as logicians say, of exhaustion, showing what disease is by showing what it is not. Every answer that is elicited strikes out a certain number of contingencies, and so circumscribes the area of what is still uncertain. Indeed, the making a diagnosis is very much like the game of "Twenty Questions," in which several people fix upon one word or thing, while another is to ascertain what that word or thing is by the answer he gets to twenty questions put to them in order. If the questions are well and methodically put, the discovery of the subject thought of is almost inevitable, however eccentric or bizarre it may be, and often a good deal within the prescribed number of questions. To take for the sake of illustration, an example from this game which I remember to have occurred. *Q.* Is it animal, vegetable or mineral? *A.* Animal. *Q.* Is it marine or terrestial? *A.* Marine. *Q.* Is it natural or artfiicial? *A.* Natural. *Q.* Is it useful or ornamental? *A.* Ornamental. Here was an animal marine production, used as an ornament in its natural state. At this stage the questioner guessed a pearl, and his guess was right; indeed, the answers to these four questions seemed to leave no alternative. See now how much each of these questions excluded, how much the first—two-thirds, we may say, of created things; how much the second—more than a half of this remaining third; then, how much the third; while the exclusion implied by the answer to the fourth was almost complete. Just such, and with such an object, should be the interrogations put in the diagnosis of disease.

Now, this principle of elimination or exhaustion gives rise to two rules with regard to the order and kind of questions put to your patients.

*a.* Put first those questions which exclude the greatest number of alternatives, that is, what is called "leading questions."

*b.* Let each of the questions be devoted exclusively to the elimination of a certain amount of those alternatives, which the previous answers have left undecided; let them apply strictly and solely to what is left unsettled, that is, do not throw away any questions on points that previous questions have set at rest.

The order and kind of questions, based on these rules, that seem to me the best, and which I commonly observe, is something as follows:

1. Where is the pain (or symptom, whatever it may be)?
2. How long has it existed?
3. Has it ever occurred before?
4. The apparent cause?
5. In what way the functions of the organ are disturbed?
6. The lædentia and juvantia?
7. Anatomical evidence?
8. Physical examination?
9. History, especially with regard to
   *a.* Inherited diathetic peculiarity, such as struma, gout, rheumatism, etc.?
   *b.* Alcoholic, or other intemperance?
   *c.* Syphilis?
   *d.* Occupation?

It would not be necessary to put all these questions in all cases, some would be settled by few of them; for example, most stomach affections would be settled by the fifth or sixth, most chest affections by the eighth; many affections would carry you on to question 9, clause *a*; delirium tremens to clause *d*; clause *c* will often be the turning point of your diagnosis; while lead palsy would carry you on to clause *d*. Often, however, you will not have to put all the questions; the answers that you will receive from some will involve an inversion of the order of the rest, and you will make a short cut to a determining point.

Rule IV.—Other points must be observed in examining your patient, besides method.

First. Be slow and deliberate; give yourselves all the advantage over, and hold on, your patient, that you will gain by deliberation, self-possession, and presence of mind. Besides, you will, by a sort of infection, induce a similar character in your patient's answers, and you will have time to deliberate on the answer you get.

Secondly. Ask one thing at a time, and let the single object of that question be as definite as possible.

Thirdly. Pin your patient to your question; do not let him wander and be irrelevant, do not let him be indefinite, do not let him be evasive. You will often be troubled with answers of all three kinds, especially from some classes of patients; the first kind you will get from old women and the garrulous, the second from the Irish, and the third from malingeres; indeed, you might name them the *responsio garrula*, the *responsio Hibernica*, and the *responsio mimotica*, respectively. I know of few things more difficult than making a prompt diagnosis out of an Irish patient; he calls his chest his stomach, and his stomach his chest; his heart is ubiquitous; if you ask him how long he has been ill, he tells you "a good bit;" and if he is better, he says he is "no worse." I speak of the lower orders of Irish only, and I mention it as a warning, that you may know what to expect, and prepare yourselves accordingly, when you hear the Irish accent.

Fourthly. Be considerate and patient, and kind in your manner. This you will find of immense advantage, not only to your patient but to yourself. You will get both kinds of evidence much better, more truthful, and more natural—both that which you will receive in answer to questions, and that which your patient will furnish you with unconsciously to himself. In timid persons, and those whose affections are of an emotional character, this point is of especial importance, and most of all in children; if you once set a child crying, your further investigation of its case is greatly embarrassed. Dr. West, in his work on diseases of children, has made some admirable observations on this point, that ought to be engraved on the memory of every one having any thing to do with the treatment of children: I will rather refer you to them than mutilate them by abbreviation.

Rule V.—In difficult cases, where you are at a loss, it is often a good plan to forego the ordinary method of interrogating, and adopt some other. One of these is to

Let the patient tell his own story in his own way. In this way sometimes things will creep out that set-questions had not elicited, or questions may be suggested which before had not been thought of, but which may reach exactly the point which was required to clear up the doubt. Another is,

Begin at the head and go downwards, as hunters "draw" a forest; pass through all the organs in a regular anatomical order, so that the state of none may elude you. In this way sometimes you may stumble upon some mischief in a part where you had little suspected it: at any rate you will be sure of meeting it where it exists, and will feel that all the organs which in your progress you leave, as it were, in your rear, may be disregarded.

Rule VI.—Do not be content till you have elicited a natural group of symptoms, but when you have once done this, assure yourself that you have attained your object. Do not let any amount of evidence in the opposite scale countervail it—the conclusiveness of such evidence is absolute, not relative; do not let any improbabilities, and deficiency of cause, any suspicion of collusion, shake your faith in the infallibility of this internal, this intrinsic evidence. No amount of sophistication can enable the uninitiated to similate the natural and self-consistent features of disease.

Rule VII.—Make yourselves what jurists call "skilled witnesses;" familiarize yourselves with all those means of diagnosis, and of enlarging and rendering more precise your knowledge of disease, which the physician of the present day finds at his command, or you may renounce all hope, either of doing justice to yourselves or your patients, or of competing with your brothers in the profession, who are familiar with the use of these invaluable instruments—instruments, the revelations of which have almost transformed the face of modern medicine. There was a time when people used to die of dropsy; nobody dies of dropsy now. Those who used to die of dropsy now die of lung, or heart, or liver, or ovarian, or kidney disease. And what has shown us this? Mainly, the stethoscope, the microscope, and the test-tube. We cannot afford to depreciate or dispise these aids, we shall be but blind guides without them; and besides, human life is too precious a thing, and our tutelage of it too solemn, to permit us, in any thing that appertains to it, to give way to prejudice, or a miserable conservative obstructiveness.

The stethoscope was despised once, and there are many now of the same class of mind who attempt to depreciate the microscope, the chemical reagent, the stethometer, the weighing machine, the ophthalmoscope, the test-paper, and the spirometer. We may safely leave them to fight their own battle. In the meantime, instead of abusing them let us use them, let us make ourselves masters of them, let us add them to our resources.

Rule VIII.—Lastly, observe for yourselves, think for yourselves, judge for yourselves. Do not constantly carry about with you, opinions that you have read as the interpreters of what you see; if you do, it will constantly hamper and shackle your minds, you will get yourselves into a habit of seeing only what others have seen, and be blind to a great deal of instruction that disease will offer you. The present generation of medical men read too much and think too little; the chronic and almost endemic malady from which we suffer is a plethora of books. There are some who can never see or discuss a case without quoting some authority; instead of seeing with their own eyes they are always seeing with the eyes of Graves, or Allison, or Andral.

Now, in literature, "authority" may be final, and the author of the Illiad and the Æneid may determine the force of a particle or the length of a syllable; but in science this is not so, and the testimony of nature must always overrule that of her interpreters. If you want to make any advance in medicine; if you would add any thing to the present stock of our knowledge; if you would receive into your minds the teachings of disease in all their simplicity and freshness, you must see and hear with your own eyes and ears, and interpret for yourselves. Vindicate to yourselves, and constantly exercise, that attribute of the best class of minds—independent and self-reliant thought; take as your own the motto of our highest scientific society, and early learn *nullius in verba magistri jurare.—Nashville Journal.*

